# Knowledge and attitudes toward recreational cannabis legalization among California residents: a population-matched questionnaire about Proposition 64

**DOI:** 10.1186/s42238-025-00304-9

**Published:** 2025-07-12

**Authors:** Daniel Ageze, Sara Baird, Thomas D. Marcotte, Ahmed Elashmawy, Angela Wong, Renee Dell’Acqua, Jill Rybar, Sarah Hacker, Alice Gold, Tom Shaughnessy, Ilene Lanin-Kettering, Linda L. Hill

**Affiliations:** 1 Herbert Wertheim School of Public Health and Longevity Science, University of California San Diego, 9500 Gilman Drive, La Jolla, CA 92093 USA; 2 Center for Medicinal Cannabis Research, Department of Psychiatry, University of California San Diego, 220 Dickinson Street, Suite B, San Diego, CA 92103 USA; 3 Quester, 6500 University Avenue Suite 205, Des Moines, IA 50324 USA

**Keywords:** Cannabis, Cannabis knowledge, Marijuana, Proposition 64, Prop 64, Cannabis legalization

## Abstract

**Background:**

Implemented in 2018, Proposition 64: The Adult Use of Marijuana Act (Prop 64; passed in 2016) legalized adult recreational cannabis use in California. This analysis of the Impact 64 study aimed to assess knowledge about Prop 64 and attitudes toward cannabis legalization by California residents.

**Methods:**

A mixed qualitative and quantitative questionnaire about cannabis and Prop 64 was completed by 4,020 current cannabis users, 523 former users, and 635 non-users who were selected from an initial pool demographically matched to the 2020 California census. Quantitative questions about Prop 64 knowledge and attitudes were selected for this sub-analysis. Chi-squared tests and nominal logistic multivariate analysis were used to assess knowledge and attitudes based on demographic characteristics and cannabis use factors.

**Results:**

Current users had a mean age of 42 years and were 59% male, and more than 90% had heard of Prop 64. The 71% of current users who described themselves as somewhat or very familiar with Prop 64 were more likely to be employed full-time (AOR 1.5, *p* < 0.001), have no minors in the household (AOR 1.4, *p* < 0.001), and live in Central California (AOR 1.3, *p* < 0.001). Awareness of specific regulations of the associated laws was low among current users, including for possession (49%), transportation (41%), and gifting (30%) of cannabis, as well as for age and possession limits. Attitudes were mixed, though current users had more positive attitudes than former and non-users for almost all measures (*p* < 0.001). Only 30% of current cannabis users, believe cannabis should be legal to use in more public places, and only half of current users felt that Prop 64 made cannabis products and cannabis acquisition safer.

**Conclusions:**

Four years after implementation, knowledge gaps around Prop 64 are widespread. Effective messaging is needed to increase awareness and bridge knowledge gaps, which can ultimately minimize public harm.

**Supplementary Information:**

The online version contains supplementary material available at 10.1186/s42238-025-00304-9.

## Background

A major turning point for cannabis legalization in the United States came in 1996 when California became the first state to legalize cannabis for medicinal use (California 2025). Since then, public support has continued to grow for the legalization of cannabis for both medicinal and recreational use, and in 2016, California Proposition 64: The Adult Use of Marijuana Act (Prop 64) legalized the use of recreational cannabis in the state of California for individuals over the age of 21 (Marchini 2016). Implemented in 2018, Prop 64 outlines the logistic and legal framework for the sale and possession of recreational cannabis products (Judicial 2025).

Remaining illegal on the federal level under the Controlled Substances Act (CSA), state-level cannabis laws often implement specific regulations and restrictions which, for example, set limits on use, prohibit interstate commerce, and create regulatory frameworks such as licensing, testing and quality control (Alcohol 2025). Cannabis legalization via state-level law has resulted in variability in these specific restrictions, but broadly, for distributors they outline licensing requirements, restrictions on transportation, advertising, packaging and labeling, and for users, they outline requirements for possession limits, where cannabis can be consumed, and impaired driving (Judicial 2025).

Proponents of recreational cannabis legalization cite multiple potential benefits, ranging from legal (for example, freeing law enforcement to pursue non-cannabis related crime, and recognition of the racial disparities in prior cannabis-related law enforcement), to safety (for example, improved personal safety while obtaining cannabis) and health (for example, consistent quality of products, and outlining locations for use that will minimize secondhand smoke exposure) (Todd 2018). Yet despite the growing public support and the increasing number of states that have legalized cannabis for medicinal and/or recreational use, there is little research about public awareness of laws surrounding cannabis legalization that could influence the real-world impact of legalization; this includes the differences between state and federal laws, and the specific regulations regarding possession limits, public use, and impaired driving. In one study done after legalization in Vermont, sixty percent of young adults correctly identified the state’s cannabis policy (legal possession for adults over age 21); correct knowledge was associated with being older, White non-Hispanic, more educated, and ever or past-month use (West et al. 2022). Research has instead focused on assessing public support of legalization, and the changing cannabis use patterns particularly among high-risk groups (Ladegard et al. 2023, Farrelly 2023). Understanding patterns of awareness among the general public may point to disparities and guide public health interventions such as educational campaigns. In 2021, the Herbert Wertheim School of Public Health and Human Longevity Science at University of California San Diego (UC San Diego) was funded by the California Department of Cannabis Control (DCC) to evaluate the impact of Prop 64 on cannabis use patterns and knowledge about cannabis legalization. This analysis from the Impact 64 study aims to assess the current state of knowledge about Prop 64 and attitudes toward cannabis legalization among California adults. For this study, we emphasized that “cannabis” use involved the use of THC-containing products, to differentiate from CBD-only cannabis products.

## Methods

Impact 64 employed a multi-phase, mixed-methods approach, involving three phases: 1) Subject Matter Expert (SME) interviews with 23 individuals, including legal professionals, medical providers, advocates, researchers, users, and dispensary representatives; 2) an exploratory questionnaire with 200 participants; and 3) a large-scale questionnaire targeting 5,000 California residents from an initial pool of more than 15,000 screened participants weighted to match the CA census demographics. This analysis focuses on selected results from the third phase. All study procedures were approved by the UC San Diego Institutional Review Board (IRB), number 808894.

### Questionnaire development

Based on findings from the Phase 1 SME interviews and Phase 2 exploratory questionnaire of 200 participants, a 25-min study questionnaire was developed in partnership with Quester, a market research firm. A mixed methods study design was utilized, incorporating multiple-choice questions and open-ended items with skip logic based on participant responses; however, only quantitative data were included in this analysis. A sample of the study questionnaire pertinent to this analysis is included in the supplemental materials.

### Participants and recruitment

Using Quester’s network of market research sampling partners, the research team used quota sampling to obtain a representative sample of Californians by gender, age, race/ethnicity, and annual household income. The 2020 California Census was used to establish quotas for each demographic category. These targeted individuals were invited online through the partner sampling organizations to complete an initial “screener” questionnaire which collected demographics and cannabis use history, with the purpose of identifying eligible participants for the full questionnaire. Participants were not aware of the study's purpose at the time of the screener questionnaire. The sampling partners provided study participants with points that could later be redeemed for cash, gift cards, and other prizes. The points provided had a total value no greater than $10. In addition to demographic quotas, inclusion criteria were residence in California, 21 years of age or above, and the ability to read English or Spanish. Those employed in the cannabis, marketing/market research, or advertising/public relations industries were excluded.

A subset of participants was directed to complete the full questionnaire, with a goal of 5000 participants broken down by cannabis use history: current cannabis users (self-identified as current user and used within the past 3 months; goal *n* = 4,000), former cannabis users (self-identified as former user and has not used in 4 or more months; goal *n* = 500), and non-users (goal *n* = 500). Target sample size was identified based on power calculations, with a larger sample size required for current users due to additional questions and subsequent analysis within this group. Selection for the full questionnaire was based on cannabis use and demographic targets within each group which were intermittently monitored, and selected participants completed the full questionnaire immediately following the screener questionnaire. Participants were excluded from the full questionnaire for exceeding use group and demographic quotas (*n* = 991), non-qualification (*n* = 7,367), or incomplete participation (*n* = 1,841). Incomplete participation was assessed using factors such as the speed of completion (too fast vs expected length), presence and quality of answers in the open-ended questions, number of incomplete fields, as well as if participants used a “center-lining” approach to answer the questionnaire. Recruitment ceased once target numbers were met.

### Data collection

The official questionnaire was launched on December 2, 2022, and remained open until February 6, 2023, when all target participants were recruited. The questionnaire was online and compatible with computer and mobile devices. No personal identifying information was collected.

### Statistical analysis

Using rake weighting (iterative proportional fitting), respondents of the screener questionnaire were weighted based on the California census for four key demographic criteria (age group, gender, race/ethnicity, annual household income). Demographic profiles of each cannabis subgroup (current, former, non-users) were identified based on this weighted screener group, establishing target demographics for each subgroup. Within each full questionnaire subgroup, participants were weighted to match each subgroup’s demographic profile to the target. As a result, the screener participant group’s demographics matched the broader California population, while each full questionnaire subgroup’s demographics match the demographics of that user group within California.

Analysis focused on questions from the Impact 64 questionnaire related to knowledge and attitudes surrounding cannabis policy and legalization and utilized weighted data which is presented except where noted. Descriptive statistics were used to explore sample characteristics, and chi-square tests were performed to examine differences across demographic and cannabis use groups. Nominal logistic multivariate analysis was utilized to evaluate associations while adjusting for demographics, including age, gender, race/ethnicity, education, income, employment, marital status, minors in household, region, age at first use, and use frequency. Statistical analysis was conducted using SPSS v. 28.0.0.0 (IBM 2024) and JMP Pro v. 17.0.0 (SAS Institute Inc n.d.). Statistical significance was assessed as *p* < 0.05.

## Results

A total of 15,208 participants completed both demographics and cannabis use history on the screener questionnaire and were assessed for eligibility for the full questionnaire. Although region was not included as a key demographic for quota sampling or weighting, the regional distribution of the screener population approximated the 2020 California census. Weighted demographics for age, gender, race/ethnicity, and annual household income otherwise exactly matched census targets. Unweighted and target Census demographics of the screener questionnaire are presented in Supplement 1. Of the initial questionnaire respondents who did not know the purpose of the study, 37% reported current cannabis use (use in the past 3 months), 30% were former users, and 33% were non-users.

The full questionnaire was completed by 5,178 participants, including 4,020 current users, 523 former users, and 635 non-users. Participants in each use group were weighted to match their target demographics set by the screener questionnaire. After weighting, demographics exactly matched the target for all use groups. Unweighted and target demographics of current users are presented in Supplement 2. The results presented below are based on the weighted data from the full questionnaire.

### Demographics

The demographics of each use group are summarized in Table 1. Among current users, the mean age is 42 years, 59% male, 38% White non-Hispanic, 82% have at least some education beyond high school, and 57% have minors in the household. The mean age of first use is 24.4 years (SD 13): 53% of current users started using cannabis before age 21, and 33% started at age 17 or younger. Twenty nine percent of current users are occasional users (3 times per week or less), 33% use between 4 times per week to daily, and 38% reported cannabis use multiple times per day. After adjustment for other demographics, with age as a continuous variable, and minors in household as a dichotomous variable (yes/no), current users were more likely than former or non-users to be younger, male, Hispanic (any race) or Black non-Hispanic (*p* < 0.001) and working full time. Univariate comparisons by cannabis use category are shown in Table 1.

**Table 1:** Demographics of the participants of the Impact 64 full questionnaire by cannabis use history

	Current users *n* = 4020 %	Former users *n* = 523 %	Non-users *n* = 635 %
**Age**			
Mean age	42 (SD 14) ^**B**,^ ^**C**^	48 (SD 16) ^**C**^	52 (SD 16)
**Gender**			
Male	59% ^**B**,^ ^**C**^	47% ^**C**^	40%
Female (ref)	41% ^**B**,^ ^**C**^	53% ^**C**^	60%
**Race/Ethnicity**			
White non-Hispanic	38% ^**B**^	44% ^**C**^	39%
Hispanic (all races)	42% ^**B**,^ ^**C**^	35% ^**C**^	30%
Black non-Hispanic	8% ^**B**,^ ^**C**^	5%	5%
Asian/Pacific Islander (ref)	11% ^**B**,^ ^**C**^	14% ^**C**^	25%
**Educational** **status**			
High school diploma or lower	18%	15%	17%
Some college or college degree	68% ^**C**^	69% ^**C**^	64%
Graduate degree (ref)	14%	15%	19%
**Annual** **Household** **income**			
Under 50 K	24% ^**C**^	26%	28%
50-100 K (ref)	28%	29%	30%
Greater than 100 k	48% ^**C**^	45%	42%
**Employment** **status**			
Employed full-time (ref)	64% ^**B**,^ ^**C**^	50%	45%
Employed part-time	13%	14%	14%
Unemployed	23% ^**B**,^ ^**C**^	35%	41%
**Marital** **status**			
Single (ref)	45% ^**B**^	49% ^**C**^	43%
Married or has partner	55% ^**B**^	51% ^**C**^	57%
**Minors** **in** **household** ^**y**^			
No minors in household (ref)	43% ^**B**,^ ^**C**^	49%	53%
Minors in household	57%	51%	47%
Age 0–6	19%	19%	15%
Age 7–12	27%	18%	15%
Age 13–17	21%	15%	15%
**Residence/Region**			
Northern region	26%	27%	24%
Central region	16%	17%	15%
Southern region (ref)	58%	57%	61%
**Age** **of** **first** **cannabis** **use**			
Mean age	24.4 (SD 13)	23.6 (SD 13)	–
**Use** **frequency**			
Occasional use (3 times per week or less)	29%	–	–
Frequent use (4 times per week to daily)	33%	–	–
Very frequent use (more than once daily)	38%	–	–

Significance was determined using bivariate analysis by comparing each cannabis user category. Annotations (^A,^ ^B,^ ^C^) show statistical significance using *p*-value < 0.05, where the user groups (columns A, B, C) differed from each other user group

### Familiarity with Prop 64

Current users reported being significantly more likely to be very or somewhat familiar with Prop 64 (73%), versus former users or non-users (55–56%, *p* < 0.001), as shown in Table 2. Few participants in all groups had never heard of Prop 64, including only 6% of current users. On multivariate analysis, current users who reported being very or somewhat familiar with Prop 64 were more likely to be employed full-time (77% vs. 65% unemployed, AOR 1.5, *p* < 0.001), have no minors in the household (67% vs 79% with minors, AOR 1.4, *p* < 0.001), and live in Central California (68% vs. 74% Northern California, AOR 1.3, *p* < 0.001) (not shown in table).

**Table 2:** Participant awareness of cannabis possession and use restrictions

	**Current** **users** **(A)** *n* = 4020 Weighted %	**Former** **users** **(B)** *n* = 523 Weighted %	**Non-users** **(C)** *n* = 635 Weighted %
*How familiar are you with Prop 64, the law that legalized* *recreational use of cannabis in California in 2016?*			
Very familiar	32%^BC^	12%	13%
Somewhat familiar	40%	44%	41%
Have heard of it, but don’t know much about it	22% ^BC^	33%	37%
Have never heard of it	6% ^BC^	10%	9%
*Which of the following are you aware are legal for adults 21* + *under the Prop 64 law?*			
Possess 1 oz. or less of recreational and a quarter of an oz. or less of concentrate	49% ^BC^	43%	40%
Transport 1 oz. or less of recreational and a quarter of an oz. or less of concentrate in the trunk of the vehicle	41% ^BC^	35%	30%
Give away 1 oz. or less of recreational and a quarter of an oz. or less of concentrate to other adults 21 +	30% ^BC^	24%	21%
Use of cannabis on private property, but not in public spaces	64%	68%	65%
*Which of the following are you aware that are true under* *the Prop 64 law?*			
A person cannot smoke cannabis in places where it is illegal to smoke tobacco	55%	57%	59%
Prop 64 does not decriminalize cannabis use for minors, but it does reduce the maximum penalty for most cannabis-related offenses to an infraction (except for manufacturing and driving under the influence of cannabis)	45% ^BC^	38% ^C^	31%
A person currently serving a sentence for a conviction of an eligible cannabis-related offense may petition the court for resentencing or dismissal of eligible convictions	44% ^BC^	36%	33%

^ABC^ Denotes statistical significance (*p* < 0.001) of that column (A, B) compared to the other columns (B, C), using Chi-squared analysis

### Awareness of possession and use restrictions

Compared to former cannabis users and non-users, current users were more likely to report being aware of the laws regarding the possession, transportation, and gifting of cannabis (49%, 41%, 30% for current users, respectively), as shown in Table 2. About two thirds of participants in all groups reported being aware that cannabis could be used on private property but not in public spaces (64–68%), and 55–59% knew that cannabis use was illegal in places where smoking tobacco use is illegal. However, awareness for all other categories was low, below 50%.

Awareness was assessed for each demographic group after correcting for other demographics (not shown in table). For all seven awareness measures listed in Table 2, awareness was more likely in White non-Hispanic participants (vs. Hispanic any race, AOR 1.3–1.6, *p* < 0.001). Those with higher educational attainment were more likely to be aware for six of the seven measures (vs. high school diploma or less, AOR 1.3–1.9, *p* < 0.001). Regarding possession, transportation, and gifting regulations, males were more likely than females to report awareness (AOR 1.2–1.5, *p* < 0.001).

### Perceived legality of cannabis use in different settings

Table 3 summarizes the perceived legality and illegality of cannabis use in various locations and settings. Overall, there were few differences between current, former, and non-user knowledge, and current user data is outlined here except where specified. Most current users were able to correctly identify legal use within one’s own home (86%) and illegal use in indoor public spaces (71%), and restaurants (66%). There was less certainty shown for other measures, as demonstrated by more equal distribution between legal/illegal responses, or by fewer respondents choosing either answer. This was particularly noticeable for ‘outdoor public space’ (21% legal, 44% illegal, correct answer illegal), and ‘in the same places where it is legal to smoke cigarettes’ (24% legal, 29% illegal, correct answer illegal).

**Table 3:** Perceived legality of cannabis use by location, among current users

	**Current** **users** *n* = 4020 Weighted %		**Former** **users** *n* = 523 Weighted %		**Non-users** *n* = 635 Weighted %	
*Under the Prop 64 law, where is it legal/illegal to use/consume cannabis?*^+^	Legal	Not legal	Legal	Not legal	Legal	Not legal
Your home (legal)	86%	3%	89%	1%	82%	2%
Someone else’s home (legal)	51%	10%	60%	5%	42%	11%
Outdoor public space (not legal)	21%	44%	28%	38%	23%	37%
Restaurant (not legal)	5%	66%	2%	77%	3%	70%
Indoor public spaces, like museums and schools (not legal)	6%	71%	2%	80%	3%	74%

^+^Participants were asked to mark which locations were legal/illegal and could select any; does not total 100%. Correct responses are noted in parentheses

The demographics of respondents who were correct for perceived legality varied with a couple notable exceptions (not shown in table). After correcting for other demographics, individuals with minors in the household were less likely to accurately identify that it is legal to use at other people’s home (43% vs 57% without minors, AOR 1.3, *p* < 0.001), not legal in restaurants (65% vs. 72% without minors, AOR 1.5, *p* < 0.001), and not legal indoor public spaces (72% vs 77%, AOR 1.8, *p* < 0.001). Male participants were also more likely than female participants to falsely report that it is legal to use cannabis in restaurants (7% vs 3%, AOR 1.9, *p* < 0.001), and indoor public spaces (7% vs. 3%, AOR 2.0, *p* < 0.001).

### Knowledge of age and possession limits

Participants were asked to enter the legal age of recreational and medicinal cannabis use as outlined in Prop 64; findings are summarized in Table 4. For the age limit, 66% of current users provided an answer, versus 45% of former users and 50% of non-users; the remainder were not sure. Of the current users who responded, 75% correctly reported the recreational age limit (21 years old). Fewer participants correctly reported the legal age for medicinal cannabis use (18 years old). Participants were also asked to report the number of grams of cannabis one can legally carry for recreational use. Current users were more likely to answer this question (37%) than former users (14%) and non-users (11%); the remainder provided no answer. Of the current users who responded, only 33% of current users accurately reported 28 g to be the legal limit for recreational use.

**Table 4:** Knowledge of Age and Possession Limits for Cannabis under Prop 64, by use type

*Under the Prop 64 law, what is the legal age for using each type of cannabis?*						
	**Current** **Users** *n* = 4020 Weighted %		**Former** **Users** *n* = 523 Weighted %		**Non-users** *n* = 635 Weighted %	
**No** **answer** **/** **Not** **sure**	34%		55%		50%	
**Gave** **an** **answer**	66%		45%		50%	
	**Medicinal**	**Recreational**	**Medicinal**	**Recreational**	**Medicinal**	**Recreational**
**18** **years**	35%	16%	45%	29%	42%	30%
**21** **years**^**+**^	54%	75%	51%	70%	54%	68%
**Other**	11%	9%	4%	1%	4%	2%
*Under the Prop 64 law, how many grams of recreational cannabis can you legally carry on your person?*						
	**Current** **Users**		**Former** **Users**		**Non-users**	
**No** **answer** **/** **Not** **sure**	63%		86%		89%	
**Gave** **an** **answer**	37%		14%		11%	
	**Recreational**					
**Under** **28** **g**	60%		72%		72%	
**28** **g** ^**+**^	33%		25%		24%	
**Over** **28** **g**	7%		3%		4%	

+ Correct response for medicinal age: 18 years. Correct age for recreational age: 21 years. Correct response for recreational dried flower: 28 g

### Attitudes towards cannabis policy and legalization

There were significant differences between current users and non-users in the attitudes towards cannabis policy and legalization which are summarized in Table 5. Current users were significantly more likely than non-users to report that the federal government should legalize cannabis in all states (59% vs 22%, *p* < 0.001), that cannabis should be legal to use in places where smoking is allowed (40% vs. 15%, *p* < 0.001), that creating new variations and new methods to consume it is a positive trend (35% vs. 8%, *p* < 0.001), and that cannabis should be legal to use in more public places (30% vs. 4%, *p* < 0.001). Current users were more likely to think that legalization improved safety of the cannabis products and the environment of acquiring cannabis (54% and 51% vs 20% and 23%, respectively, *p* < 0.001). Non-users were significantly more likely than current users to think smoking cannabis will lead to or worsen lung and breathing problems (52% vs. 23%, *p* < 0.001), that legalizing cannabis will lead to increased use by minors (40% vs. 14%, *p* < 0.001), cannabis use will lead to the increased use of other drugs (37% vs. 8%, *p* < 0.001), and that using cannabis will result in use that one can’t stop (28% vs. 8%, *p* < 0.001.).

**Table 5:** Attitudes of current, former, and non-users towards cannabis policy and legalization

**Strongly** **agree** **with** **the** **following** **statement:**	Current users (A) *n* = 4020 Weighted %	Former users (B) *n* = 523 Weighted %	Non-users (C) *n* = 635 Weighted %
The federal government should legalize cannabis in all states	59% ^BC^	52% ^C^	22%
The legalization of cannabis improved the safety of cannabis products	54% ^C^	51% ^C^	20%
The legalization of cannabis improved personal safety when making cannabis purchases	51% ^C^	49% ^C^	23%
Cannabis should be legal to use in places where smoking is allowed	40% ^BC^	30% ^C^	15%
Creating new variations and new methods to consume it is a positive trend	35% ^BC^	26% ^C^	8%
Cannabis should be legal to use in more public places	30% ^BC^	13% ^C^	4%
Smoking cannabis will lead to or worsen lung and breathing problems	23% ^BC^	39% ^C^	52%
Legalizing cannabis will lead to increased use by minors	14% ^BC^	28% ^C^	40%
Cannabis use will lead to the use of other drugs	8% ^BC^	17% ^C^	37%
Using cannabis will result in use that one can’t stop	8% ^BC^	10% ^C^	28%
Using cannabis during pregnancy is safe	7% ^BC^	2%	1%

^ABC^ Denotes statistical significance (*p* < 0.001) of that column (A or B) compared to the other associated column (B or C), using Chi-squared analysis

### Changes in purchasing and patterns since passage of Prop 64

The impact of Prop 64 on participant purchasing and use patterns of cannabis are summarized in Table 6. Most respondents reported increased convenience, with only 10% traveling farther distances to obtain cannabis. Sixty-four percent of participants felt safer obtaining cannabis than they did prior to legalization, with only 4% feeling less safe. One third (35%) of participants reported spending more on cannabis after passage of Prop 64. A similar percentage of participants reported using more medicinal (32%) and recreational (36%) cannabis than they were before Prop 64. Participants who reported increased use of recreational cannabis after passage of Prop 64 were more likely to be male (32% vs 23% female AOR 1.4, *p* < 0.001), have a graduate degree (34% vs 27% high school diploma or less, AOR 1.6, *p* < 0.001), be employed (31% vs 19% not employed, AOR 1.4, *p* < 0.001), have minors in the household (36% vs 23% none, AOR 1.5, *p* < 0.001), and started using cannabis under the age of 17 (34% vs 23% of those who started over age 45, AOR 6.5, *p* < 0.001) (not shown in table).

**Table 6:** Changes in cannabis purchasing and use patterns among current users since passage of Proposition 64

	Current users *n* = 3078* Weighted %		Current users *n* = 3078 Weighted %
**Safety** **of** **purchasing** **cannabis**		**Cost**	
More safe	64%	Spend more	35%
Equally safe	32%	Spend the same	49%
Less safe	4%	Spend less	17%
**Overall** **use** **amount** **–** **medicinal**		**Travel** **distance** **to** **obtain** **cannabis**	
Use more	34%	Travel farther	10%
Use the same	53%	Travel the same	37%
Use less	14%	Travel shorter	39%
**Overall** **use** **amount—recreational**		Switched to delivery	13%
Use more	37%		
Use the same	51%		
Use less	12%		

^*^Unweighted n; number of participants who started using cannabis before passage of Prop 64

## Discussion

Implemented in 2018, Prop 64 legalized the recreational use of cannabis in California state. Among participants demographically matched to California residents, overall self-reported awareness of Prop 64 was high across all groups, with 71% of current users reporting being somewhat or very familiar with Prop 64. Yet, despite the general awareness about Prop 64, knowledge gaps were demonstrated in all facets of the associated laws.

While there is no research demonstrating public knowledge and familiarity with specific cannabis legalization laws, several tobacco studies show knowledge gaps when new laws are passed (Dai et al. 2021, Tan et al. 2022, Chellamuthu et al. 2023). The qualitative SME interviews done in Phase 1 of the Impact 64 study suggested that consumer outreach regarding specifics of the Prop 64 laws had been limited (not published), and this analysis of awareness and knowledge supports that finding. In the current analysis, fewer than half of participants were aware of possession and transportation laws, while fewer than one third of participants were aware that gifting limited amounts of cannabis was now legal. This low knowledge of key facets of Prop 64, even among current users, warrants attention and public outreach.

Knowledge gaps are a concern due to the potential impact of law enforcement involvement due to inadvertent illicit cannabis use. For example, while most current users were correctly aware that it was legal to use cannabis in their home or someone else’s home, 21% of current users believe it is legal to use cannabis in outdoor public spaces, and many current users were unsure about the legality of cannabis use outside the home. Although multivariate analysis showed no knowledge differences by race, Black cannabis users are more likely to be arrested for cannabis use even in post-legalization states, despite cannabis use rates that are largely equal between racial groups (Gunadi et al. 2022). Efforts to increase awareness and bridge the knowledge gap among BIPOC communities may help address racial disparities. Additionally, participants with minors in the household were less likely to correctly identify legal settings; this should be a topic of future research to mitigate potential health (exposure to secondhand smoke) and legal (involvement of child protection services) consequences.

Knowledge regarding the legal age and possession limits were also low even among current users. Although distinguishing between medicinal and recreational cannabis use is necessary, the varying age for medicinal and recreational use can also by itself create a confusion among the public. Additionally, state-by-state legalization has led to great heterogeneity of age and possession limits. One 2021 study demonstrated a legal range of 1–2.5 oz for flowers, 3.5–15 g for concentrates, and even greater differences for edibles (Steuart 2023, Pacula et al. 2021). Moreover, some state laws also limit sales of cannabis by doses per transaction, again with a wide range, from 560 (Alaska) to 2,283 (Michigan); since no potency limits exist in any state, the measure of ‘doses’ is additionally determined by comparing median product potencies (Pacula et al. 2021). Given these complexities, it is not surprising that few participants correctly identified possession limits under Prop 64. Thus, it crucial that some level of standardization should be made to minimize the risk of misinformation and subsequent legal consequences.

Attitudes toward cannabis policy and legalization were mixed, with former users and non-users expressing fewer positive and more negative attitudes compared to current users. Participants of all use types were not inclined to support expanding legal use to more public places. Among current users, 63% reported feeling safer obtaining cannabis following the passage of Proposition 64, while 54% strongly agreed that cannabis legalization improved the safety of cannabis products. Given that a primary objective of cannabis legalization efforts was to enhance safety, these proportions appear unexpectedly low. Sixty percent of current users strongly agree that the federal government should legalize cannabis in all states, consistent with the 57–68% of U.S. adults who support full cannabis legalization in 2023–24 (Gallup 2022, Pew 2024).

Despite broad awareness and reported familiarity, knowledge of specific facets of Prop 64 was mixed among current cannabis users in California. Ongoing public education campaigns to clarify Prop 64 regulations will help on a personal level (avoid accidentally violating regulations) and public health (prevent smoke exposure in public locations). Mixed attitudes among all participants reflect the complexity of public opinion. Future research should bring focus to this nuance, monitor trends in knowledge, and follow up the efficacy of public outreach and intervention.

A strength of this study is the large initial sample size that was matched to the California 2020 census demographics, which allows for reasonable generalization of study findings to the cannabis use group populations of California. Limitations include the exclusion of individuals under age 21, which limits insights into younger users. Response bias may contribute to over- or under-reporting, particularly for self-reporting of illicit or high-risk behavior, though participation was anonymous, and this is less likely to impact the current analysis on knowledge and attitudes. Similarly, question phrasing such as “Which of the following are you aware are legal” implies legality and may result in overestimation of positive response; this bias, however, reinforces findings of low overall knowledge. The online format of the questionnaire disproportionately excludes populations without access to technology. Finally, as a cross-sectional study, causation and changes in findings over time cannot be assessed.

## Conclusion

There are widespread knowledge gaps about Prop 64 among Californian adults of all cannabis user types. Closing the knowledge gap, particularly among current cannabis users, may help minimize harm from legal and health consequences of unintentionally illicit cannabis use. There are likely multiple contributing factors, and interventional efforts can target different demographic groups based on specific campaign goals. Creating and executing well-informed messaging will take collaboration with the consumers of various backgrounds, the cannabis industry, and various stakeholders including those in academia and public spheres.

## Supplementary Information

Supplementary Material 1.

Supplementary Material 2.

Supplementary Material 3.

## Data Availability

The datasets used and/or analyzed during the current study may be available from the corresponding author on reasonable request.

## Abbreviations

AOR: Adjusted odds ratio
CSA: Controlled Substance Act
DCC: Department of Cannabis Control
IRB: Institutional Review Board
Prop 64: Proposition 64
SME: Subject Matter Expert
THC: Delta 9 Tetrahydrocannabinol
UC San Diego: University of California San Diego
