# Supplementary material for: Knowledge and attitudes toward recreational cannabis legalization among California residents: a population-matched questionnaire about Proposition 64
Source: J Cannabis Res. 2025 Jul 12;7:42. doi: 10.1186/s42238-025-00304-9 (PMC12254984; doi:10.1186/s42238-025-00304-9)
Supplement: Supplementary file 1 — Supplementary Material 1. [file 42238_2025_304_MOESM1_ESM.docx]

**Supplement 1:** Demographics of population by California census/target vs. demographics of unweighted/actual study population for screener questionnaire

|  | **Target** | **Actual Screener**  **(n=15,309)** |  | **Target** | **Actual Screener**  **(n=15,309)** |
| --- | --- | --- | --- | --- | --- |
| **Age** | | | **Region** | | |
| 21-34 | 28% | 29% | **Northern Region** | | |
| 35-44 | 18% | 19% | Superior California | 8% | 9% |
| 45-54 | 17% | 17% | North Coast | 2% | 2% |
| 55+ | 37% | 34% |  |  |  |
| **Gender** |  |  | San Francisco Bay Area | 18% | 15% |
| Male | 49% | 48% |  |  |  |
| Female | 51% | 52% | **Central region** | | |
| **Ethnicity/Race** | | | Northern San Joaquin Valley | 5% | 4% |
| White non-Hispanic | 40% | 38% |  |  |  |
| Black non-Hispanic | 6% | 6% | Central Coast | 6% | 5% |
| Hispanic (all races) | 36% | 37% | Southern San Joaquin Valley | 7% | 7% |
| Asian/Pacific Islander | 16% | 15% | **Southern region** | | |
| Other | 2% | 2% | Inland Empire | 12% | 14% |
| **Annual Household Income** | | |  |  |  |
| <$50k | 26% | 26% | Los Angeles | 25% | 27% |
| $50k - $99k | 29% | 33% | Orange | 8% | 8% |
| $100k | 45% | 41% | San Diego - Imperial | 9% | 9% |
