# Supplementary material for: Knowledge and attitudes toward recreational cannabis legalization among California residents: a population-matched questionnaire about Proposition 64
Source: J Cannabis Res. 2025 Jul 12;7:42. doi: 10.1186/s42238-025-00304-9 (PMC12254984; doi:10.1186/s42238-025-00304-9)
Supplement: Supplementary file 2 — Supplementary Material 2. [file 42238_2025_304_MOESM2_ESM.docx]

**Supplement 2:** Demographics of census-weighted/target current users vs. demographics of unweighted/actual current users

|  | **Target** | **Actual**  **Current Users**  **n=4,020** |  | **Target** | **Actual**  **Current Users**  **n=4,020** |
| --- | --- | --- | --- | --- | --- |
| **Age** | | | **Region** | | |
| 21-34 | 39% | 41% | **Northern Region** | | |
| 35-44 | 22% | 26% | Superior California | 8% | 9% |
| 45-54 | 18% | 16% | North Coast | 2% | 2% |
| 55+ | 21% | 17% |  |  |  |
| **Gender** |  |  | San Francisco Bay Area | 18% | 15% |
| Male | 59% | 50% |  |  |  |
| Female | 41% | 50% | **Central region** | | |
| **Ethnicity/Race** | | | Northern San Joaquin Valley | 5% | 4% |
| White non-Hispanic | 38% | 39% |  |  |  |
| Black non-Hispanic | 8% | 11% | Central Coast | 6% | 5% |
| Hispanic (all races) | 42% | 39% | Southern San Joaquin Valley | 7% | 7% |
| Asian/Pacific Islander | 10% | 9% | **Southern region** | | |
| Other | 2% | 3% | Inland Empire | 12% | 11% |
| **Annual Household Income** | | |  |  |  |
| <$50k | 24% | 34% | Los Angeles | 25% | 31% |
| $50k - $99k | 28% | 36% | Orange | 8% | 7% |
| $100k | 48% | 30% | San Diego - Imperial | 9% | 9% |
