# Supplementary material for: Knowledge and attitudes toward recreational cannabis legalization among California residents: a population-matched questionnaire about Proposition 64
Source: J Cannabis Res. 2025 Jul 12;7:42. doi: 10.1186/s42238-025-00304-9 (PMC12254984; doi:10.1186/s42238-025-00304-9)
Supplement: Supplementary file 3 — Supplementary Material 3. [file 42238_2025_304_MOESM3_ESM.pdf]

Initial Screener Questionnaire

S0Industry

Do you or does anyone in your household work in any of the following types of businesses or occupations? Please select all that apply.

Opt. #	Option Text
1	Advertising/Public Relations
2	Financial Services
3	Food/Beverage
4	Marketing/Marketing Research
5	Real Estate/Construction
6	Sales/Sales Promotion
7	Cannabis Industry
8	None of the above

S1aZipCode

What is your residential zip code?

S1bState

In which state do you live?

Opt. #	Option Text
1	Alabama
2	Alaska
3	Arizona
4	Arkansas
5	California
6	Colorado
7	Connecticut
8	Delaware
9	District of Columbia
10	Florida
11	Georgia
12	Hawaii
13	Idaho
14	Illinois
15	Indiana
16	Iowa
17	Kansas
18	Kentucky
19	Louisiana

20	Maine
21	Maryland
22	Massachusetts
23	Michigan
24	Minnesota
25	Mississippi
26	Missouri
27	Montana
28	Nebraska
29	Nevada
30	New Hampshire
31	New Jersey
32	New Mexico
33	New York
34	North Carolina
35	North Dakota
36	Ohio
37	Oklahoma
38	Oregon
39	Pennsylvania
40	Rhode Island
41	South Carolina
42	South Dakota
43	Tennessee
44	Texas
45	Utah
46	Vermont
47	Virginia
48	Washington
49	West Virginia
50	Wisconsin
51	Wyoming
52	Outside of the US

S2aAge
What is your age?

S3Gender
What is your gender?

Opt. #	Option Text
1	Male
2	Female
3	Genderqueer or non-binary
4	Agender
5	Not specified above
6	Prefer not to answer

S4aHispanic

Are you of Spanish, Hispanic, or Latino background or origin? This includes Mexican, Mexican American, Puerto Rican, Cuban, and all other Spanish, Hispanic, or Latino origins.

Opt. #	Option Text
1	Yes
2	No
3	Prefer not to answer

S4bRace

Are you...? Select all that apply.

Opt. #	Option Text
1	White/Caucasian
2	Black/African American
3	American Indian/Native American
4	Asian
5	Pacific Islander
6	Some other race
7	Prefer not to answer

S5aPersonalIncome

What is your personal income (not including others in your household) before taxes?

Opt. #	Option Text
1	Less than $25,000
2	$25,000 to $49,999
3	$50,000 to $74,999
4	$75,000 to $99,999
5	$100,000 to $124,999
6	$125,000 to $149,999
7	$150,000 to $249,999
8	$250,000 or more

S5bHHIncome

And what is your total annual household income (including others in your household) before taxes?

Opt. #	Option Text
1	Less than $25,000
2	$25,000 to $49,999
3	$50,000 to $74,999
4	$75,000 to $99,999
5	$100,000 to $124,999
6	$125,000 to $149,999
7	$150,000 to $249,999
8	$250,000 or more

D2HHSize

How many people are living or staying at your current address? (Include yourself and any other adults or children who are living or staying at this address for at least two months)

Opt. #	Option Text
1	1
2	2
3	3
4	4
5	5
6	6
7	7
8	8 or more
9	Prefer not to answer

D2aHHComposition

And just to be sure I understand, thinking of who are living or staying at your current address, are any of them … ?

Opt. #	Option Text
1	Under age 6
2	7 to 12 years old
3	13 to 17 years old
4	My adult children age 18+
5	My parents or in-laws
6	Spouse or partner
7	My grandchildren
8	Grandparents
98	Other family member (aunts, uncles, cousins)

9	Roommate(s)
99	Prefer not to answer

D2bNumberKids

How many of those living in your household are children under the age of 18?

Opt. #	Option Text
1	0
2	1
3	2
4	3
5	4
6	5
7	6 or more
8	Prefer not to answer

S7Usage
Which of the following statements best describes you?

Please note: THC (tetrahydrocannabinol) is the substance in cannabis products that is responsible for the ‘high’ or ‘stoned’ feeling when using cannabis. Pure CBD products do not contain THC.

Opt. #	Option Text
1	I currently use or consume **cannabis that contains THC** (in any form)
2	I do not currently use or consume **cannabis that contains THC** (in any form), but I have in the past
3	I have never used or consumed **cannabis that contains THC** (in any form)

Ask if Current or Former User
S8LastConsumed
When was the last time you used or consumed <u>cannabis that contains THC</u>, in any form?

Opt. #	Option Text
1	Today
2	Within the past week
3	Within the past month
4	Within the past 2-3 months
5	Within the past 4-6 months
6	Within the past 7-12 months

7	More than 12 months ago

S9UseFrequency

Ask Current Users (S7=1): Approximately how often do you use or consume cannabis, in any form, that contains THC?
Ask Past Users (S7=2): Approximately how often did you use or consume cannabis, in any form, that contains THC **in the past**?

Opt. #	Option Text
1	Multiple times a day
2	Once a day
3	4-6 times a week
4	1-3 times a week
5	1-2 times a month
6	Once every 2 to 3 months
7	Once every 4 to 6 months
8	Less than every 6 months

Q1AgeUse
At what age did you start regularly/consistently using cannabis?

Q56ChangeTravel
How has the distance you travel to obtain cannabis changed since it became legal to possess/use in California in 2018?
Since it became legal to possess/use in California in 2018:

Opt. #	Option Text
1	I travel longer distances to obtain cannabis
2	I travel shorter distances to obtain cannabis
3	I travel the same distance to obtain cannabis
4	I switched to delivery

Q58SafetyAfter
Since it became legal to possess/use in California in 2018, have you felt a change in how you feel about **your physical/personal safety** when it comes to obtaining cannabis?

Opt. #	Option Text
1	I feel safer
2	I feel less safe
3	No change

Q33Familiarity
Type: Radio
How familiar are you with Prop 64, the law that legalized recreational use of cannabis in California in 2016?

Opt. #	Option Text
1	Very familiar
2	Somewhat familiar
3	Have heard of it, but don’t know much about it
4	Have never heard of it

Q37InfluencedUse
How did Prop 64 influence your own use of cannabis?

Opt. #	Option Text
1	It made it **much more likely** for me to use cannabis
2	It made it **somewhat more likely** for me to use cannabis
3	It made it **neither more likely nor less likely** for me to use cannabis

4	It made it **somewhat less likely** for me to use cannabis
5	It made it **much less likely** for me to use cannabis

Q41Knowledge

Which of the following are **TRUE** regarding Prop 64? Select all that apply.

Opt. #	Option Text
1	It's legal to smoke cannabis in the car as a passenger
2	You can get a DUI for driving under the influence of cannabis
3	Individual cities can set their own regulations regarding driving after using cannabis
4	Any container of cannabis inside a moving vehicle must be unopened and sealed, just like alcohol
99	None of the above

Q44Amount_Different

Under the Prop 64 law, how many grams of each type of cannabis can you legally carry on your person?

Medical cannabis: _____grams
Recreational cannabis: _____grams

- Not Sure

Q47Age_Different

Under the Prop 64 law, what is the legal age for using each type of cannabis?

Medical cannabis: _____years-old
Recreational cannabis: _____years-old

- Not Sure

Q48aPlacesLegal

Under the Prop 64 law, where is it **legal** to use/consume cannabis? Select all that you feel to be legal; please do not select an item if you are not sure whether or not it is legal.

Opt. #	Option Text
1	Your home
2	Someone else's home
3	Outdoor public space
4	Restaurant
5	Indoor public spaces, like museums and schools
6	In the presence of children under 18
7	In the same places where it is legal to smoke cigarettes

99	None of the above

Q48bPlacesNotLegal
Under the Prop 64 law, where is it **not legal** to use/consume cannabis? Select all that you feel are not legal; please do not select an item if you are not sure whether or not it is legal.

Opt. #	Option Text
1	Your home
2	Someone else's home
3	Outdoor public space
4	Restaurant
5	Indoor public spaces, like museums and schools
6	In the presence of children under 18
7	In the same places where it is legal to smoke cigarettes
99	None of the above

Q50Awareness1
Which of the following are you aware are legal for adults 21+ under the Prop 64 law? Select all that apply.

Opt. #	Option Text
1	Possess 1 ounce or less of recreational cannabis and a quarter of an ounce or less of cannabis concentrate
2	Use cannabis on private property, but not in public places, such as bars or restaurants
3	Give away 1 ounce or less of recreational cannabis and a quarter of an ounce or less of cannabis concentrate to other adults 21 years and older without compensation
4	Transport 1 ounce or less of recreational cannabis and a quarter of an ounce or less of cannabis concentrate cannabis in the trunk of the vehicle
99	None of the above

Q51Awareness2
Which of the following are you aware that are true under the Prop 64 law? Select all that apply.

Opt. #	Option Text
1	A person cannot smoke cannabis in places where it is illegal to smoke tobacco
2	Drivers and passengers cannot smoke or ingest cannabis products in a moving vehicle
3	Prop 64 does not decriminalize cannabis use for minors, but it does reduce the maximum penalty for most cannabis-related offenses to an infraction (except for manufacturing and driving under the influence of cannabis)
4	A person currently serving a sentence for a conviction of an eligible cannabis-related offense may petition the court for resentencing or dismissal of eligible convictions
99	None of the above

Q52aCannabisPerceptionsAgree
Please select the statements about cannabis that you, personally, **strongly agree with**.

Randomize

Opt. #	Option Text
1	Cannabis should be legal to use in places where smoking is allowed
2	Cannabis should be legal to use in more public places
3	The federal government should legalize cannabis in all states
4	Cannabis use will lead to the use of other drugs
5	Legalizing cannabis will lead to increased use by minors
6	Using cannabis during pregnancy is safe
7	Smoking cannabis will lead to or worsen lung and breathing problems
8	Using cannabis will result in use that one can't stop
9	The legalization of cannabis improved the safety of cannabis products
10	The legalization of cannabis improved personal safety when making cannabis purchases
11	Creating new variations and new methods to consume it is a positive trend
99	None of the above

Q61SpendFrequency

How has Prop 64 impacted the amount of money you **spend** on cannabis each month?

Opt. #	Option Text
1	Spending more
2	Spending less
3	Spending the same amount

Q63aUseFrequencyMedicinal

How has **Prop 64** impacted how frequently you use **medicinal** cannabis?

Opt. #	Option Text
1	I use medicinal cannabis more often
2	I use medicinal cannabis less often
3	I use medicinal cannabis as often as before

Q63bUseFrequency

How has **Prop 64** impacted how frequently you use **recreational** cannabis?

Opt. #	Option Text
1	I use recreational cannabis more often
2	I use recreational cannabis less often
3	I use recreational cannabis as often as before

D1Marital

What is your marital status?

Opt. #	Option Text
1	Single, never married
2	Living with partner
3	Married
4	Widowed
5	Divorced/Separated
6	Prefer not to answer

D3Education

What is the highest degree or level of school you have completed?

Opt. #	Option Text
1	Less than 9th grade
2	9th to 12th grade, no diploma
3	Regular High School Diploma
4	GED or alternative credential
5	Occupational trade program
6	Some college credit, but no degree
7	Associate's degree (for example: AA, AS)
8	Bachelor's degree (for example: BA, BS)
9	Master's degree (for example: MA, MS, MEng, MEd, MBA)
10	Professional degree (for example: MD, DDS, DVM, LLB, JD)
11	Doctorate degree (for example: PhD, EdD)

D4Employment

Which of the following best describes your current employment status?

Opt. #	Option Text
1	Employed – full-time
2	Employed – part-time
3	Self-Employed – full-time
4	Self-Employed – part-time
5	Retired
6	Student – full-time
7	Student – part-time
8	Military
9	Full-time parent, homemaker
10	Not currently employed
11	Prefer not to answer
